# Peripheral blood T-lymphocyte subsets are potential biomarkers of disease severity and clinical outcomes in patients with ulcerative colitis: a retrospective study

**DOI:** 10.1186/s12876-023-02769-5

**Published:** 2023-04-27

**Authors:** Bailu Geng, Xueli Ding, Xiaoyu Li, Hua Liu, Wenjun Zhao, Haihong Gong, Zibin Tian, Jing Guo

**Affiliations:** 1 Department of Gastroenterology, the Affiliated Hospital of Qingdao University, Qingdao, 266003 Shandong China; 2 Department of Respiratory and Critical Care Medicine, the Affiliated Hospital of Qingdao University, Qingdao, China

**Keywords:** T-lymphocyte subsets, Biomarkers, Ulcerative colitis, Prognosis

## Abstract

**Background:**

Ulcerative colitis (UC) is considered an immune-mediated disease. The disorder of T-lymphocyte subsets plays an important role in the pathogenesis of UC. The aim of this study was to evaluate the significance of peripheral blood T-lymphocyte subsets in assessing disease severity and predicting clinical outcomes in UC patients.

**Methods:**

The retrospective case-control study was performed in 116 UC patients with active disease and 90 healthy controls (HC). The UC patients included were followed up for 180 days. Analyses of *t*-test, Spearman’s correlation coefficient, multivariable Cox regression analysis, receiver operating characteristic (ROC) curves and cumulative survival analysis were done.

**Results:**

The UC patients had lower proportions of CD4^+^T cells (42.85%±9.77% vs 45.71%±7.94%, *P*=0.021) and higher proportion of CD8^+^T cells (27.88%±8.86% vs 25.00%±6.47%, *P*=0.008) than HC. The severely active UC patients had higher proportion of CD3^+^HLA-DR^+^ T cells (8.83%±6.55% vs 2.80%±1.55%, *P*<0.001; 8.83%±6.55% vs 4.06%±5.01%, *P*<0.001) and CD8^+^T cells (31.35%±8.49% vs 26.98%±7.98%, *P*=0.029; 31.35%±8.49% vs 25.46%±9.15%, *P*=0.003) than mild and moderate group, whereas lower proportion of CD4^+^CD25^+^T cells (2.86%±1.35% vs 3.46%±1.07%, *P*=0.034) than mild group and CD4^+^T cells (40.40%±9.36% vs 44.73%±10.39%, *P*=0.049) than moderate group. The area under the curve (AUC) of CD3^+^HLA-DR^+^ T cells for assessing severely active UC was 0.885, with the cut-off value of 5.33%. The sensitivity was 76.32% and specificity was 89.74%. The combination of CD3^+^HLA-DR^+^ T cells and CRP had stronger assessment value with AUC of 0.929. The AUC of CD8^+^T cells, CD4^+^/CD8^+^ ratio and CD4^+^CD25^+^T cells for assessing disease severity was 0.677, 0.669 and 0.631 respectively. Within the 180 days follow-up, 24 patients (20.69%) had UC-related readmission or surgery, with higher proportion of CD3^+^HLA-DR^+^ T cells (10.66%±9.52% vs 3.88%±2.56%, *P*=0.003) and CD8^+^T cells (31.19%±10.59% vs 27.01%±8.20%, *P*=0.039) than those without readmission and surgery. The proportion of CD3^+^HLA-DR^+^ T cells was the independent predictor of UC-related readmission or surgery (HR=1.109, *P*=0.002). The AUC of CD3^+^HLA-DR^+^ T cells for predicting readmission or surgery was 0.796 with the cut-off value of 5.38%. UC patients with CD3^+^HLA-DR^+^T cells proportion>5.38% had a shorter time to readmission or surgery (log-rank test, *P*<0.001).

**Conclusions:**

The combination of CD3^+^HLA-DR^+^T cells and CRP may be potential biomarker of disease severity in UC patients. The high proportion of CD3^+^HLA-DR^+^T cells may be associated with an increased risk of readmission or surgery in UC patients.

## Background

Ulcerative colitis (UC) is one form of inflammatory bowel disease (IBD) characterized by chronic recurrent inflammation of colon and rectum. UC is considered a progressive disease and approximately 20% UC patients may require hospitalization because of severe disease activity and 10%-15% are at risk of colectomy during the disease course [1]. The long-standing intestinal inflammation increases the risk of dysplasia and colonic carcinogenesis of UC patients [2].Therefore, maintaining long-term remission and avoiding disease recurrence is of great importance in reducing complications and improving prognosis in UC patients. These goals emphasize the need to monitor the disease severity and predict upcoming exacerbations of disease for UC patients timely and accurately in order to choose the optimal treatment regimen [3, 4]. Colonoscopy combined with pathological biopsy is considered the crucial standard for evaluating the severity of UC [5]. However, the invasive procedure of endoscopy may cause painful experience and is associated with a risk of perforation, especially in severely active UC patients, limiting its application in frequent disease activity monitoring [6]. Therefore, non-invasive biomarkers are needed for serially assessing disease severity and detecting an imminent flare. There have been several studies explored the value of serum and fecal inflammatory markers, such as C-reactive protein (CRP), erythrocyte sedimentation rate (ESR) and fecal calprotectin, for assessing disease activity and prognosis in UC patients [5, 7–9]. But the sensitivities and specificities of these markers still remain highly controversial, as well as the high cost and lack of standard quality control of fecal calprotectin also limit their use in clinic [9]. Therefore, practical and accurate biomarkers for disease severity and prognostic assessment are urgently needed in UC patients.

UC is considered an immune-mediated disease, in particular the widespread and successful use of biological agents targeting immune abnormalities of UC patients in recent years [10, 11]. The adaptive immune system, especially the dysregulated T lymphocyte response is thought to play the key role in the chronic intestinal inflammation of UC patients [2]. Several researches have reported that there were dysregulated T-lymphocyte subsets not only in colitis models [12] but also in UC patients [13, 14]. The proportion of T-lymphocyte subsets were correlated with the inflammatory activity [15] and the disease course of UC patients [16]. Given their important role in the pathogenesis of UC, exploring T-lymphocyte subsets as non-invasive biomarkers for disease severity and prognostic assessment may highly improve the accuracy, compared with general inflammatory markers. However, the associations between specific T-lymphocyte subsets and disease severity of UC are still not clearly. And there remains a lack of study to quantify the clinical outcomes of UC patients with specific T-lymphocyte subsets. Therefore, our study aimed to explore the compositional changes of T-lymphocyte subsets among UC patients and healthy controls and the correlation of T-lymphocyte subsets and disease severity. The association between T-lymphocyte subsets and clinical outcomes was also explored.

## Methods

### Ethics approval

The study was conducted in accordance with the Declaration of Helsinki (as revised in 2013). This study was approved by the Ethics Committee of the Affiliated Hospital of Qingdao University (NO. QYFY WZLL 27363). The need for written informed consent was waived by the Ethics Committee of the Affiliated Hospital of Qingdao University due to retrospective nature of the study. The study protocol has been registered in the Chinese Clinical Trial Registry (identifier: ChiCTR2200065103).

### Participants

This retrospective case-control study included 116 patients with confirmed diagnoses of UC and 90 healthy controls (HC) admitted to the Affiliated Hospital of Qingdao University from January 2018 to December 2021. The inclusion criteria of UC group were as follows: (I) A confirmed diagnosis of UC based on the combination of clinical, laboratory, endoscopic and histopathological evaluation [3]; (II) All patients were in the active phase of the disease with the Mayo score≥3 [10]; (III) All patients had been diagnosed with UC previously and followed-up in our hospital; (IV) Complete data and information. The exclusion criteria were as follows: (I) The patients complicated with hematological diseases, malignant tumors or autoimmune diseases which were not related to UC; (II) The patients unavailable for follow‐up. 90 randomly selected healthy controls who underwent endoscopy for physical examination in our hospital were included. All healthy controls received endoscopy assessments. The individuals with ulcerative colitis and other immune-mediated diseases were excluded.

Participants were followed up for 180 days from the time of T-lymphocyte subsets measurement. The clinical outcomes were defined as UC-related readmission due to recurrence and underwent bowel surgery due to failure of medical therapy.

### Diagnostic criteria

The disease severity of UC patients was assessed by the Mayo score according to stool frequency, rectal bleeding, findings on endoscopy and physician’s global assessment, and further categorized into the mild activity (3-5 scores), moderate activity (6-10 scores) and severe activity (11-12 scores) [10]. The endoscopic disease activity was graded by the Ulcerative Colitis Endoscopic Index of Severity (UCEIS) according to vascular pattern (0-2 scores), bleeding (0-3 scores), erosion and ulcers (0-3 scores) [17]. The disease extent was assessed using the Montreal classification and classified into E1 (proctitis), E2 (left-sided UC) and E3 (extensive UC) [3].

### Data collection

Peripheral blood T-lymphocyte subsets of participates were recorded containing the proportion of CD3^+^T cells, CD4^+^T cells, CD8^+^T cells, CD4^+^/CD8^+^ ratio, CD4^+^CD25^+^T cells and CD3^+^HLA-DR^+^T cells. The demographic and clinical data were collected including sex, age, body mass index (BMI), disease duration, smoking history, alcohol consumption history, history of appendectomy, extraintestinal manifestation and the treatment which the UC patients were taking. The laboratory data were recorded including the counts of white blood cells (WBC), neutrophils (NE), lymphocytes (LYM), platelets (PLT), hemoglobin (Hb), albumin (Alb), erythrocyte sedimentation rate (ESR) and C-reactive protein (CRP). All demographic, clinical and laboratory data were obtained within 7 days of T-lymphocyte subsets measurement.

### Statistical analysis

Quantitative variables with normal distribution were presented as mean ± standard deviation, while as median and interquartile range with non-normal distribution. Qualitative variables were presented as numbers and percentages. The Kolmogorov Smirnov test was used to test detect the normality of the data. Comparisons of the clinical baseline data between UC patients and healthy controls were used by *t*-test (or Mann-Whitney U test as appropriate), comparisons among mild, moderate and severe subgroups of UC patients were used by one-way analysis of variance with least significant difference post-hoc test for continuous variables and chi-square test (or Fisher’s exact test as appropriate) for categorical variables. The correlations between the proportion of T-lymphocyte subsets and the Mayo score and UCEIS were detected using Spearman’s rank correlation coefficient. The efficacy of T-lymphocyte subsets for assessing severe activity, UC-related readmission and surgery were evaluated by receiver operating characteristic (ROC) curve analysis and the cut-off value were confirmed with maximum sensitivity and specificity. The independent prognostic factors associated with UC-related readmission and surgery were determined using the multivariate Cox proportional hazard regression analysis, with the hazard ratio (HR) and 95% confidence interval (CI) describing the relative effect. The impact of high proportion of CD3^+^HLA-DR^+^T cells on the clinical outcomes of UC was evaluated with the Kaplan-Meier (K-M) curves and log-rank test. SPSS 26.0 software (IBM) was used to analyze data and *P*-value<0.05 was defined as statistical significance.

## Results

### Baseline characteristics of UC patients and healthy controls

A total of 116 UC patients with active disease and 90 HC were included in our study. Among the 116 UC patients, 69 men (59.5%) and 47 women (40.5%) were included with the average age of 46.75±16.47 year-old. There was no statistical difference in gender and age between UC patients and HC (*P*>0.05). The UC patients were divided into mildly active group including 37 patients, moderately active group including 41 patients and severely active group including 38 patients according to Mayo score. The demographic and clinical characteristics of participates were shown in Table 1.

**Table 1:** Baseline characteristics of ulcerative colitis (UC) patients and healthy controls (HC)

Characteristics	UC (*n*=116)	Mild activity (*n*=37)	Moderate activity (*n*=41)	Severe activity (*n*=38)	HC (*n*=90)	P value ^a^	*P* value ^b^	*P* value ^c^	*P* value ^d^
Sex						0.377	0.058	0.674	0.012^*^
Male	69	20	31	18	48				
Female	47	17	10	20	42				
Age (years)	46.75±16.47	50.24±13.23	43.27±17.71	47.11±17.54	50.06±10.09	0.077	0.063	0.408	0.300
BMI (kg/m^2^)	21.71±3.47	23.03±2.42	22.69±3.73	19.38±2.87	25.51±3.39	<0.001^*^	0.626	<0.001^*^	<0.001^*^
Disease duration(months)	56.36±63.08	54.54±61.81	67.87±67.08	45.72±59.33	—	—	0.352	0.545	0.121
Smoking history	13(11.21%)	2(5.41%)	4(9.76%)	7(18.42%)	16(17.78%)	0.179	0.678	0.153	0.338
Alcohol consumption history	17(14.66%)	2(5.41%)	8(19.51%)	7(18.42%)	19(21.11%)	0.226	0.091	0.153	0.902
History of appendectomy	8(6.90%)	2(5.41%)	4(9.76%)	2(5.26%)	1(1.11%)	0.081	0.179	0.978	0.676
Extraintestinal manifestations	13(11.21%)	2(5.41%)	6(14.63%)	5(13.16%)	—	—	0.268	0.430	0.850
Montreal classification						—	0.336	<0.001^*^	0.001^*^
E1	5	4	1	0	—				
E2	17	7	10	0	—				
E3	94	26	30	38	—				
Treatment						—	0.005^*^	<0.001^*^	0.035^*^
5-aminosalicylic acid	45	23	19	3	—				
Steroids	28	1	14	13	—				
Immunosuppressants	3	1	1	1	—				
Biological agents	40	12	16	12	—				
WBC (×10^9^/L)	7.02±2.74	5.79±1.61	7.18±2.47	8.03±3.41	5.93±1.44	<0.001^*^	0.020^*^	<0.001^*^	0.153
NE (×10^9^/L)	4.48±2.22	3.36±1.26	4.58±1.93	5.45±2.75	3.10±0.96	<0.001^*^	0.011^*^	<0.001^*^	0.064
LYM (×10^9^/L)	1.89±0.81	1.87±0.59	1.93±0.74	1.88±1.05	2.18±0.60	0.005^*^	0.765	0.971	0.792
PLT (×10^9^/L)	304.04±105.03	245.41±62.95	302.54±102.42	362.76±110.03	224.19±46.28	<0.001^*^	0.009^*^	<0.001^*^	<0.001^*^
Hb (g/L)	113.28±26.88	127.35±19.56	120.44±25.61	91.84±20.90	139.20±14.02	<0.001^*^	0.174	<0.001^*^	<0.001^*^
Alb (g/L)	35.99±6.56	40.54±2.90	37.88±5.22	29.51±5.32	40.56±3.00	<0.001^*^	0.013^*^	<0.001^*^	<0.001^*^
ESR (mm/60min)	20.44±20.28	9.78±8.32	20.09±21.32	31.20±22.06	—	—	0.016^*^	<0.001^*^	<0.001^*^
CRP (mg/L)	13.38±21.91	1.71±1.92	10.95±21.64	27.37±25.27	—	—	0.035	<0.001^*^	<0.001^*^
Clinical Outcome									
readmission and surgery	24(20.69%)	2(5.41%)	3(7.32%)	19(50.00%)	—	—	0.730	<0.001^*^	<0.001^*^

*UC* ulcerative colitis, *HC* healthy controls, *BMI* body mass index, *WBC* white blood cells, *NE* neutrophils, *LYM* lymphocytes, *PLT* platelets, *Hb* hemoglobin, *Alb* albumin, *ESR* erythrocyte sedimentation rate, *CRP* C-reactive protein.

^*^P<0.05 was considered statistically significant.

*P* value ^a^, comparing UC and HC

*P* value ^b^, comparing mildly active subgroup and moderately active subgroup

*P* value ^c^, comparing mildly active subgroup and severely active subgroup

*P* value ^d^, comparing moderately active subgroup and severely active subgroup

Compared with HC, the body mass index (BMI) (*P*<0.001), lymphocytes (LYM) count (*P*=0.005), hemoglobin (Hb) (*P*<0.001) and albumin (Alb) levels (*P*<0.001) were lower in UC patients, whereas the white blood cells (WBC) (*P*<0.001), neutrophils (NE) (*P*<0.001) and platelets (PLT) (*P*<0.001) counts were higher. There was no significant difference in smoking history, alcohol consumption and history of appendectomy between UC patients and HC (*P*>0.05). Compared among three subgroups of UC patients, the severely active UC patients had lower BMI (*P*<0.001), Hb (*P*<0.001), and Alb levels (*P*<0.001), as well as higher PLT counts (*P*<0.001), CRP (*P*<0.001) and ESR level (*P*<0.001) than mildly and moderately active group. Moreover, there were more patients with extensive disease extent in the severely active UC patients compared with the mildly and moderately active group (*P*<0.001, *P*=0.001). The severely active UC patients had higher WBC (*P*<0.001) and NE counts (*P*<0.001) than the mildly active group.

### T-lymphocyte subsets distribution in UC patients compared with healthy controls

Compared with HC, there were lower proportions of CD4^+^T cells (*P*=0.021) and CD4^+^/CD8^+^ ratio (*P*=0.025), as well as higher proportion of CD8^+^T cells (*P*=0.008) in UC patients. Compared among three subgroups of UC patients, the difference in T-lymphocyte subsets composition was more pronounced in severely active UC patients, with higher proportions of CD8^+^T cells (*P*=0.029, *P*=0.003) and CD3^+^HLA-DR^+^T cells (*P*<0.001, *P*<0.001) than mildly and moderately active group. Moreover, the severely active UC patients had lower proportions of CD4^+^CD25^+^T cells (*P*=0.034) than mildly active group and lower proportions of CD4^+^T cells (*P*=0.049) than moderately active group. There was no statistical difference of proportions of CD3^+^T cells, CD4^+^T cells , CD8^+^T cells, CD4^+^CD25^+^T cells and CD3^+^HLADR^+^T cells between mildly and moderately active UC patients (Table 2).

**Table 2:** Distribution of T-lymphocyte subsets in ulcerative colitis (UC) patients and healthy controls (HC)

Variables	UC (*n*=116)	Mild activity(*n*=37)	Moderate activity (*n*=41)	Severe activity (*n*=38)	HC (*n*=90)	*P* value ^a^	*P* value ^b^	*P* value ^c^	*P* value ^d^
CD3^+^T cells (%)	74.75±8.21	73.96±9.23	74.22±7.22	76.10±8.22	74.76±6.90	0.998	0.890	0.264	0.314
CD4^+^T cells (%)	42.85±9.77	43.27±9.18	44.73±10.39	40.40±9.36	45.71±7.94	0.021^*^	0.508	0.059	0.049^*^
CD8^+^T cells (%)	27.88±8.86	26.98±7.98	25.46±9.15	31.35±8.49	25.00±6.47	0.008^*^	0.436	0.029^*^	0.003^*^
CD4^+^/CD8^+^ ratio	1.75±0.81	1.78±0.77	2.02±0.91	1.43±0.61	2.00±0.77	0.025^*^	0.176	0.050	0.001^*^
CD4^+^CD25^+^T cells (%)	3.14±1.24	3.46±1.07	3.11±1.23	2.86±1.35	3.12±1.74	0.927	0.211	0.034^*^	0.355
CD3^+^HLADR^+^T cells (%)	5.22±5.47	2.80±1.55	4.06±5.01	8.83±6.55	4.60±2.56	0.278	0.256	<0.001^*^	<0.001^*^

*UC* ulcerative colitis, *HC* healthy controls.

^*^P<0.05 was considered statistically significant.

*P* value ^a^, comparing UC and HC

*P* value ^b^, comparing mildly active subgroup and moderately active subgroup

*P* value ^c^, comparing mildly active subgroup and severely active subgroup

*P* value ^d^, comparing moderately active subgroup and severely active subgroup

### T-lymphocyte subsets are associated with the disease severity of UC patients

**Fig. 1:**
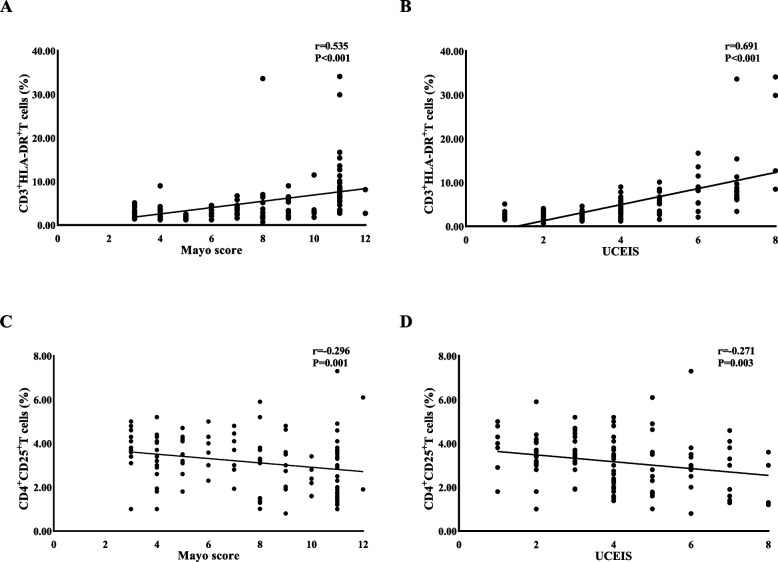
The correlation between the proportions of T-lymphocyte subsets and the disease severity of UC patients. **A**: The correlation between the proportion of CD3^+^HLA-DR^+^ T cells and the Mayo score. **B**: The correlation between the proportion of CD4^+^CD25^+^ T cells and the Mayo score. **C**: The correlation between the proportion of CD3^+^HLA-DR^+^ T cells and the UCEIS. **D**: The correlation between the proportion of CD4^+^CD25^+^ T cells and the UCEIS. Correlation was determined using Spearman’s correlation coefficient

We then assessed the correlation between T-lymphocyte subsets and the disease severity and endoscopic activity of UC patients. Both Mayo score and UCEIS showed positive correlation with the proportion of CD3^+^HLA-DR^+^T cells (*r*=0.535, *P*<0.001; *r*=0.691, *P*<0.001), and negative correlation with the proportion of CD4^+^CD25^+^T cells (*r*=-0.296, *P*=0.001; *r*=-0.271, *P*=0.003) (Figure 1).

### The value of T-lymphocyte subsets in assessing disease severity of UC patients

**Fig. 2:**
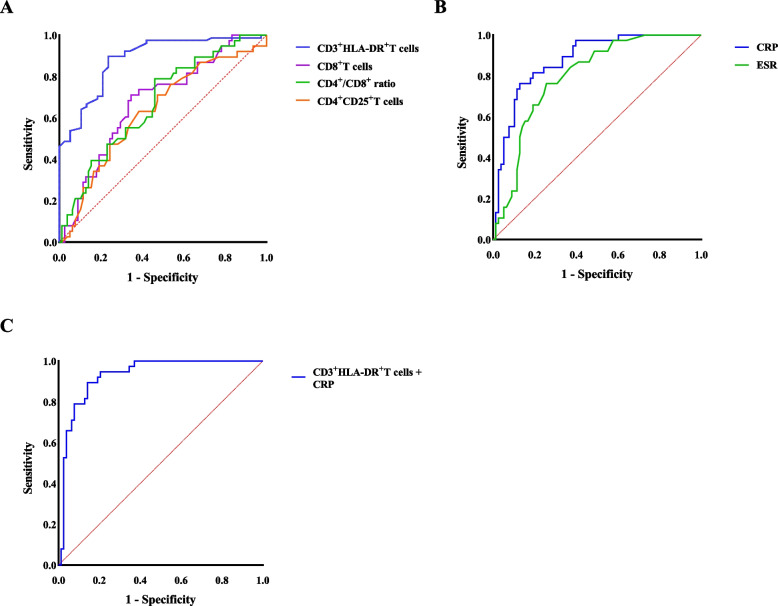
ROC curves of T-lymphocyte subsets, ESR and CRP for assessing disease severity of UC patients. **A**: ROC curves of T-lymphocyte subsets (CD3^+^HLA-DR^+^T cells, CD8^+^T cells, CD4^+^/CD8^+^ ratio and CD4^+^CD25^+^T cells). **B**: ROC curves of CRP and ESR. **C**: ROC curve of the combination of CD3^+^HLA-DR^+^T cells and CRP. ROC, receiver operating characteristic; ESR, erythrocyte sedimentation rate; CRP, C-reactive protein

In order to analyzed the value of T-lymphocyte subsets proportion in assessing disease severity of UC patients, ROC analysis was performed on the UC patients (Figure 2A). On ROC of CD3^+^HLA-DR^+^T cells, the area under the curve (AUC) for predicting severe UC was 0.885 (*P*<0.001). The cut-off value of CD3^+^HLA-DR^+^T cells proportion was 5.33%, with the sensitivity of 76.32%, and the specificity of 89.74%. Meanwhile, the efficacy of CD3^+^HLA-DR^+^T cells for assessing disease severity was higher than CRP and ESR levels in UC patients, with the AUC of 0.878 (sensitivity 76.32%, specificity 87.18%, *P*<0.001) and 0.797(sensitivity 76.32%, specificity 74.36%, *P*<0.001) respectively (Figure 2B). The sensitivities and specificities of CD8^+^T cells at the cut-off value of >27.03%, CD4^+^/CD8^+^ ratio<1.73 and CD4^+^CD25^+^T cells<3.05% for predicting severe UC were shown in Table 3, with the AUC of 0.677 (*P*=0.002), 0.669 (*P*=0.003) and 0.631(*P*=0.022) respectively. The proportions of CD3^+^T cells and CD4^+^T cells did not show the value in assessing the disease severity of UC patients (*P*>0.05).

**Table 3:** Area under ROC curves, cut-off value, sensitivity and specificity of T-lymphocyte subsets in assessing the disease severity of UC patients

Variables	AUC	95%CI	*P* value	Cut-off value	Sensitivity (%)	Specificity (%)
CD8^+^T cells	0.677	0.575-0.779	0.002	27.03%	71.05	65.38
CD4^+^/CD8^+^ ratio	0.669	0.568-0.771	0.003	1.73	53.85	78.95
CD4^+^CD25^+^T cells	0.631	0.523-0.740	0.022	3.05%	61.54	63.16
CD3^+^HLA-DR^+^T cells	0.885	0.822-0.948	<0.001	5.33%	76.32	89.74
ESR	0.797	0.716-0.878	<0.001	16.50 mm/h	76.32	74.36
CRP	0.878	0.815-0.941	<0.001	9.30 mg/L	76.32	87.18
CD3^+^HLA-DR^+^T cells and CRP	0.929	0.882-0.976	<0.001	0.24	89.47	85.90

*UC* ulcerative colitis, *ESR* erythrocyte sedimentation rate, *CRP* C-reactive protein, *ROC* receiver operating characteristic, *AUC* area under the curve, *CI* confidence interval.

We further explored the efficacy of CD3^+^HLA-DR^+^T cells combined with CRP level in assessing the disease severity. Binary logistic regression model was performed with disease severity as the dependent variable, CD3^+^HLA-DR^+^T cells proportion and CRP level as the independent variables. The combined predicted probability was calculated according to the model, which was used to plot the ROC curve of CD3^+^HLA-DR^+^T cells proportion combined with CRP level. The combination of these two indicators had stronger efficacy for predicting severe UC, with the AUC of 0.929, the sensitivity of 89.47%, and the specificity of 85.90% (*P*<0.001) (Figure 2C).

### T-lymphocyte subsets are associated with the clinical outcomes of UC patients

We further explore whether peripheral blood T-lymphocyte subsets were associated with UC-related readmission due to recurrence and surgery. There were 24 (20.69%) patients required readmission or underwent surgery during the follow-up of 180 days. The patients experiencing UC-related readmission or surgery had higher baseline proportion of CD3^+^HLA-DR^+^T cells (*P*=0.003) and CD8^+^T cells (*P*=0.039). Other factors associated with UC-related readmission or surgery included higher levels of Mayo score (*P*<0.001) and PLT (*P*<0.001), as well as lower levels of Hb (*P*<0.001), Alb (*P*<0.001) and BMI (*P*<0.001) (Table 4). We further conducted multivariate cox regression analysis to test these baseline variables as predictors for UC-related readmission or surgery and found that the proportion of CD3^+^HLA-DR^+^T cells (HR 1.109, 95% CI 1.039-1.183, *P*=0.002) and BMI (HR 0.823, 95% CI 0.708-0.957, *P*=0.011) remained the independent predictors for readmission or surgery (Table 5).There were no obvious associations between the proportions of CD3^+^T cells, CD4^+^T cells, CD4^+^/CD8^+^ ratio and CD4^+^CD25^+^T cells with the poor clinical outcomes in UC patients (*P*>0.05).

**Table 4:** Clinical characteristics of UC patients according to clinical outcomes

Variables	UC-related readmission and surgery (*n*=24)	Non- readmission and non-surgery (*n*=92)	*P* value
Sex			0.117
Male	11	59	
Female	13	34	
Age (years)	42.63±17.72	47.83±16.05	0.169
BMI(kg/m^2^)	19.24±3.10	22.36±3.28	<0.001^*^
Disease duration (months)	50.15±58.81	57.98±64.35	0.590
Smoking history	4(16.67%)	9(9.78%)	0.465
Alcohol consumption history	5(20.83%)	12(13.04%)	0.342
History of appendectomy	1(4.17%)	7(7.61%)	0.690
Extraintestinal manifestations	3(12.50%)	10(10.87%)	0.730
Montreal classification			0.153
E1	0	5	
E2	1	16	
E3	23	71	
Disease severity			<0.001^*^
Mild activity	2	35	
Moderate activity	3	38	
Severe activity	19	19	
Treatment			0.105
5-aminosalicylic acid	5	40	
Steroids	9	19	
Immunosuppressants	1	2	
Biological agents	9	31	
WBC(×10^9^/L)	7.82±3.55	6.81±2.47	0.107
NE(×10^9^/L)	5.19±3.06	4.29±1.92	0.178
LYM(×10^9^/L)	1.88±0.63	1.90±0.85	0.936
PLT(×10^9^/L)	384.88±105.43	282.96±94.65	<0.001^*^
Hb(g/L)	93.92±24.23	118.33±25.29	<0.001^*^
Alb(g/L)	29.69±6.59	37.63±5.50	<0.001^*^
ESR(mm/60min)	24.18±15.18	19.47±21.37	0.313
CRP(mg/L)	20.15±15.38	11.61±23.06	0.089
CD3^+^T cells (%)	76.71±7.72	74.25±8.30	0.193
CD4^+^T cells (%)	41.47±10.74	43.21±9.53	0.440
CD8^+^T cells (%)	31.19±10.59	27.01±8.20	0.039^*^
CD4^+^/CD8^+^ ratio	1.59±0.82	1.80±0.80	0.217
CD4^+^CD25^+^T cells (%)	3.10±1.40	3.15±1.20	0.865
CD3^+^HLA-DR^+^T cells (%)	10.36±9.42	3.88±2.57	0.003^*^

*UC* ulcerative colitis, *BMI* body mass index, *WBC* white blood cells, *NE* neutrophils, *LYM* lymphocytes, *PLT* platelets, *Hb* hemoglobin, *Alb* albumin, *ESR* erythrocyte sedimentation rate, *CRP* C-reactive protein.

^*^P<0.05 was considered statistically significant.

**Table 5:** Multivariate cox proportional hazard regression analysis of clinical outcomes

Variables	HR	95%CI	*P* value
BMI (kg/m^2^)	0.823	0.708~0.957	0.011^*^
Mayo score	1.061	0.820~1.374	0.652
PLT (×10^9^/L)	1.003	0.999~1.008	0.116
Hb (g/L)	1.002	0.983~1.021	0.827
Alb (g/L)	0.917	0.830~1.014	0.093
CD8^+^T cells (%)	0.998	0.950~1.048	0.933
CD3^+^HLA-DR^+^T cells (%)	1.109	1.039~1.183	0.002^*^

*BMI* body mass index, *PLT* platelets, *Hb* hemoglobin, *Alb* albumin, *HR* hazard ratio, *CI* confidence interval.

^*^P<0.05 was considered statistically significant.

**Fig. 3:**
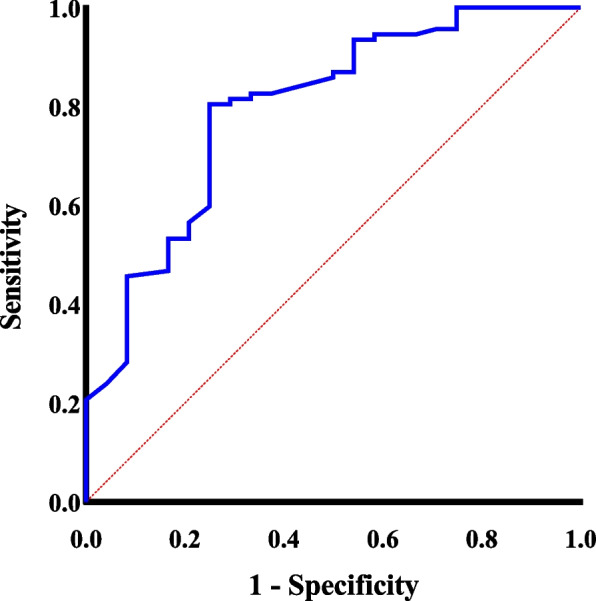
ROC curve of CD3^+^HLA-DR^+^T cells for predicting readmission or surgery of UC patients. ROC, receiver operating characteristic

**Fig. 4:**
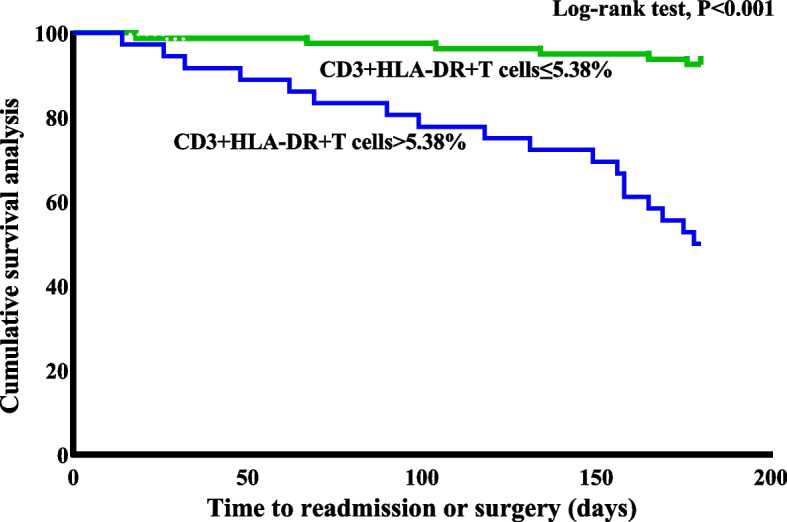
Kaplan-Meier curve of time to readmission or surgery in UC patients with high (>5.38%) and normal (≤5.38%) proportion of CD3^+^HLA-DR^+^T cells

In addition, we conducted ROC curve analysis to explore potential cut-off value of CD3^+^HLA-DR^+^T proportion for predicting clinical outcomes. The data showed CD3^+^HLA-DR^+^T cells proportion >5.38% has predicted the UC-related readmission or surgery with the AUC of 0.796 (sensitivity 75.00%, specificity 80.43%, *P*<0.001) (Figure 3). Furthermore, based on the ROC analysis, patients were divided into high and normal CD3^+^HLA-DR^+^T proportion groups according to the cut-off value of 5.38%. Then the K-M survival curve analysis showed that UC patients with CD3^+^HLA-DR^+^T cells proportion>5.38% had a shorter time to readmission or surgery (145 *vs.*175 days, log-rank test, *P*<0.001) (Figure 4).

## Discussion

There is a varying severity during the disease course in UC patients. Our study showed 32.76% of the UC patients suffered from severe disease activity, which is higher than the percentage reported in previous studies [18]. 20.69% of the UC patients experienced readmission due to disease recurrence or surgery. Early identification of severely active UC is of great importance for developing effective treatment in time to prevent life-threatening complications [19].

UC is thought to be the result of a dysregulated immune system and T lymphocytes are the main contributors [20]. As an autoimmune disease, the immunoregulatory network of UC patients could be affected by many factors, such as heredity, medications, flora and infection. All of these factors contribute to the occurrence, progression, activity and prognosis of UC through dynamic change of T lymphocytes. In another word, it is the dysregulated T lymphocytes mainly contribute to UC, no matter what factors affected or resulted in the disorder of T lymphocytes. So, the main aim of our study was to explore the clinical evaluative and predictive value of T-lymphocyte subsets in UC patients, regardless of the inducement. Therefore, the medications UC patients were taking do not affect the clinical evaluation value of T-lymphocyte subsets on disease severity and clinical outcomes. Meanwhile, several relevant studies also found that the effect of medications on lymphocytes had no impact on exploring changes of T lymphocytes in UC patients [21–23]. This study has clarified the significant changes of T-lymphocyte subsets in UC patients compared with HC which was mainly characterized by lower proportions of CD4^+^T cells and higher proportion of CD8^+^T cells. Furthermore, severely active UC patients displayed higher proportions of CD8^+^T cells and CD3^+^HLA-DR^+^T cells, as well as lower proportions of CD4^+^T cells and CD4^+^CD25^+^T cells compared with mildly and moderately active UC patients. This confirmed that the disorder of T lymphocyte response was more serious as the disease progressed. The proportions of CD3^+^HLA-DR^+^T cells and CD4^+^CD25^+^T cells both were correlated with the clinical and endoscopic severity of UC. Furthermore, the higher proportions of CD3^+^HLA-DR^+^T cells and CD8^+^T cells, as well as the lower proportions of CD4^+^CD25^+^T cells and CD4^+^/CD8^+^ ratio showed the value for assessing severely active UC. It was noteworthy that CD3^+^HLA-DR^+^T cells had a higher value for evaluating disease severity than the routine-used inflammatory indicators CRP and ESR. The combination of CD3^+^HLA-DR^+^T cells and CRP levels further improved the efficacy in evaluating the disease severity and there has been no relevant report regarding the combination of T-lymphocyte subsets and serum inflammatory markers in UC patients which need more studies to confirm.

Human leukocyte antigen D-related (HLA-DR) is a late marker of T lymphocyte activation [24]. The activation makers of T lymphocytes such as HLA-DR appear upon encountering the cognate antigen in the secondary lymphoid organs, which promotes the effector T lymphocytes mobilized from circulation to the inflammatory intestinal mucosa [25]. The present study found that there were higher proportions of CD3^+^HLA-DR^+^T cells in severely active UC patients and the elevated level of CD3^+^HLA-DR^+^T cells was a biomarker of severe UC. Several studies have also demonstrated that the proportion of HLA-DR^+^ T cells was significantly increased in UC patients and was positively related to disease activity [13, 22]. Moreover, HLA-DR^+^ T cells were also positively correlated with the systemic and mucosal inflammatory markers, such as CRP and fecal calprotectin [13, 22, 25]. All of these results highlighted the role of T lymphocyte activation in the disorder of immune response and inflammation of UC patients. That is, severe intestinal inflammation is related to the activation of T lymphocyte which is ready to enter the inflammatory gut mucosa [22]. We sought to analyze the reason for these results. On the one hand, activated T lymphocytes attack the targets in the gut mucosa of UC patients, causing destruction of intestinal epithelial cells, resulting in the damage of the mucosal barrier and then inducing inflammation [26]. On the other hand, the inflammation of gut mucosa, which may caused by intestinal microbiota dysbiosis, promotes activation of T lymphocytes by increasing the traffic of dendritic cells, which carry antigens from intestinal mucosa to lymph nodes [27]. That is, the inflammatory lesion of gut mucosa promotes T lymphocytes activation, and meanwhile this activation causes more severe inflammation of intestine. Therefore, the increased activation of blood T lymphocytes in UC may be related to the local intestinal inflammation [22] and the proportion of CD3^+^HLA-DR^+^T cells may reflect the disease severity of UC. This may explain why the severely active UC patients had higher proportion of CD3^+^HLA-DR^+^ T cells than mildly and moderately active group. Our study further analyzed the value of CD3^+^HLA-DR^+^T cells in assessing disease severity in UC. The patients with the proportion of CD3^+^HLA-DR^+^T cells>5.33% indicated a severe stage of disease*,* which may need step-up treatment for induction and maintenance of remission. Moreover, we found the efficacy of CD3^+^HLA-DR^+^T cells in assessing disease severity may be further improved by combining CRP.

A further important finding of the present study is the correlation between the proportion of CD3^+^HLA-DR^+^T cells and the poor clinical outcomes in UC patients. We found the proportion of CD3^+^HLA-DR^+^T cells was an independent risk factor of readmission and surgery for UC patients. The K-M survival analysis further demonstrated the impact of increased proportion of CD3^+^HLA-DR^+^T cells for requirements of readmission or surgery. Patients with the proportion of CD3^+^HLA-DR^+^T cells>5.38% were more likely to underwent the UC-related readmission or surgery, which need more frequent follow-up and adjust the treatment timely in order to reduce the risk of relapse and surgery. Dulic *et al.* [13]showed the proportion of HLA-DR^+^T cells is associated with the duration of remission in IBD patients. Smids *et al.* [15]found the specific T lymphocyte subsets were associated with a complicated disease course and the composition of T lymphocyte subsets of IBD patients recovered comparable to HC during follow-up with the disease course into inactive phase. All of these studies indicate the specific T lymphocyte subsets may be the biomarker of clinical outcomes in UC patients and imply whether the normalization of T cells is a more reliable prognostic marker for disease course than clinical remission, which need large prospective cohort studies to confirm.

The intestinal inflammation in UC has been traditionally contributed to abnormal immune response of CD4^+^T cells to luminal antigen [28, 29]. Previous studies demonstrated the lower baseline CD4^+^T cells in UC patients was related to active disease and more future complications [15]. The present study found UC patients, especially patients with severe disease activity, had lower proportion of CD4^+^T cells than HC. However, the value of CD4^+^T cells for assessing disease severity and predicting clinical outcomes was not showed in our study although similar results have been reported previously [16]. The reasons for this result could be the heterogeneity of CD4^+^T cells, which comprise varying proportions of highly differentiated subsets [2]. CD8^+^T cells have drawn increased attention in recent years. Several colitis models indicated that CD8^+^T cells-mediated destruction of intestinal epithelial cells led to the damage of the mucosal barrier, and then activated CD4^+^T cells and other immune cells, ultimately caused the intestinal inflammation [26, 30, 31]. Previous evidence demonstrated that the proportion of CD8^+^T cells was related to disease activity and had prognostic value for disease course in UC patients [16, 21]. In this study, severely active UC patients had higher proportion of CD8^+^T cells. Although the proportion of CD8^+^T cells>27.03% predicted the severely active UC, the sensitivity and specificity of this indicator was not high in our study. More studies are needed to explore the potential of CD8^+^T cells as a biomarker of assessing disease severity for UC patients. The study found that UC patients had a reduced CD4^+^/CD8^+^ ratio than HC, which indicated the immune function disorder [32], and the ratio exhibited a decreasing trend as the disease severity progressed. Meanwhile, our study showed the CD4^+^/CD8^+^ ratio of HC is approximately 2:1, which is consistent with previous studies [33].

CD4^+^CD25^+^ regulatory T (Treg) cells play pivotal roles in the maintenance of normal gut homeostasis, suppressing abnormal immune responses by directly inhibiting the activation and effector function of T cells and inducing various anti-inflammatory cytokines [11, 23]. This study showed the proportion of CD4^+^CD25^+^T cells was correlated with the clinical and endoscopic scores and UC patients with the proportion of CD4^+^CD25^+^T cells<3.05% were indicative of severe disease activity. However, the evaluation value of CD4^+^CD25^+^T cells was not high, which need further studies to explore whether it could be clinically useful as indicator of disease severity. Several studies have also shown that depletion of Treg cells in peripheral blood was correlated with the disease activity of UC, which was consistent with our results, whereas they were increased in the intestinal mucosa [2, 34, 35]. This may due to circulating Treg cells were recruited to lamina propria to suppress inflammation while the newly generated Treg cells were still not sufficient [15, 34].

This study has several strengths. To our knowledge, this was the first study exploring the association between peripheral blood proportion of T-lymphocyte subsets and severely active UC, as well as its prognostic value. Moreover, the indicators of peripheral blood T-lymphocyte subsets which we analyzed in this study were easily obtained and tested. This enables T-lymphocyte subsets detection to be applied widely as an evaluation indicator of disease severity in UC patients and may guide clinicians timely adjust the treatment. Nevertheless, there were some limitations. This was a single-center retrospective study that inevitably had a selective bias. And the follow-up time was relatively short. Therefore, a prospective and multi-center research is needed to further explore and confirm these results.

## Conclusion

This study showed the proportion of specific T-lymphocyte subsets, such as CD3^+^HLA-DR^+^T cells, CD8^+^T cells, CD4^+^/CD8^+^ ratio and CD4^+^CD25^+^T cells may be the promising biomarkers of severe disease activity in UC patients. The combination of CD3^+^HLA-DR^+^T cells and CRP levels is more informative in assessing the disease severity. The high proportion of CD3^+^HLA-DR^+^T cells may be associated with an increased risk of readmission and surgery in UC patients.

## Data Availability

The datasets generated during and/or analyzed during the current study are available from the corresponding author on reasonable request.
